# European Monitoring Systems and Data for Assessing Environmental and Climate Impacts on Human Infectious Diseases

**DOI:** 10.3390/ijerph110403894

**Published:** 2014-04-09

**Authors:** Gordon L. Nichols, Yvonne Andersson, Elisabet Lindgren, Isabelle Devaux, Jan C. Semenza

**Affiliations:** 1 European Centre for Disease Prevention and Control, Tomtebodavägen 11A, Solna, 17183 Stockholm, Sweden; E-Mails: isabelle.devaux@ecdc.europa.eu (I.D.); jan.semenza@ecdc.europa.eu (J.C.S.); 2 Public Health England, 61 Colindale Avenue, London NW9 5EQ, UK; E-Mail: gordon.nichols@phe.gov.uk; 3 Norwich Medical School, University of East Anglia, Norwich Research Park, Norwich, Norfolk NR4 7TJ, UK; 4 Faculty of Medicine, University of Thessaly, Larissa 41110, Greece; 5 Swedish Institute for Communicable Disease Control, 17182 Solna, Sweden; E-Mail: yvonne.m.andersson@gmail.com; 6 Institute of Environmental Medicine, Karolinska Institutet, 17177 Stockholm, Sweden; E-Mail: elisabet.ak.lindgren@gmail.com

**Keywords:** surveillance systems, climate change, environmental surveillance, geographic information systems, environmental epidemiology, infectious diseases, outbreaks

## Abstract

Surveillance is critical to understanding the epidemiology and control of infectious diseases. The growing concern over climate and other drivers that may increase infectious disease threats to future generations has stimulated a review of the surveillance systems and environmental data sources that might be used to assess future health impacts from climate change in Europe. We present an overview of organizations, agencies and institutions that are responsible for infectious disease surveillance in Europe. We describe the surveillance systems, tracking tools, communication channels, information exchange and outputs in light of environmental and climatic drivers of infectious diseases. We discuss environmental and climatic data sets that lend themselves to epidemiological analysis. Many of the environmental data sets have a relatively uniform quality across EU Member States because they are based on satellite measurements or EU funded FP6 or FP7 projects with full EU coverage. Case-reporting systems for surveillance of infectious diseases should include clear and consistent case definitions and reporting formats that are geo-located at an appropriate resolution. This will allow linkage to environmental, social and climatic sources that will enable risk assessments, future threat evaluations, outbreak management and interventions to reduce disease burden.

## 1. Introduction

Communicable disease epidemiology is closely linked to pathogen ecology, environmental and social determinants, economic factors, access to care, as well as the state of country development [1]. This has historically been mirrored in the different epidemics and new threats that have challenged humanity over time [2]. In today’s world the development of our societies and the changes of environmental and global systems are happening at such an unprecedented scale and rapid rate that they will pose new challenges to the surveillance of infectious disease threats and the development of adaptive measures [3]. Climate change has been shown to have and to continue to have both direct and indirect effects on communicable diseases, often in combination with other drivers, such as increased global travel and trade [4,5,6]. It will therefore become more and more important to prepare for projected climate change impacts, both internationally and in Europe [4], as some novel infections have the potential to spread widely and cause substantial morbidity and mortality. Public health actions are needed to prepare for the health impacts of climate change, particularly the infectious diseases ones [6,7]. Although the impacts are predicted to be higher in developing countries than in developed ones [8], it is thought that there will still be significant impacts in Europe [9]. Mapping is important in the investigation and measurement of these changes [10], and a variety of analytical approaches are possible [11]. The impacts of climate change on infectious diseases are particularly focused on vulnerable groups [12], but intervening on these groups has proven to be difficult at best [13,14].

Climate change manifests itself locally, regionally and globally, with altered patterns of temperature, precipitation, storms and winds reflecting the complex changes resulting from the slow increase in global temperatures that reflect the impact of increased greenhouse gases [6]. The frequency, duration, and intensity of heat waves have increased across Europe, and the last decade was the warmest ever recorded [15]. Climate change may impact infectious diseases in different ways [5]. Some of these impacts include an upward movement of tick vectors into higher latitude and altitude and a shift in the transmission of other vector-borne diseases. Food and water borne diseases are also susceptible to climate change because dispersion, transport, fate and environmental exposure pathways of these pathogens are intricately linked to local climate and weather conditions, although interventions may contribute more to change in the future than climate change.

**Figure 1:**
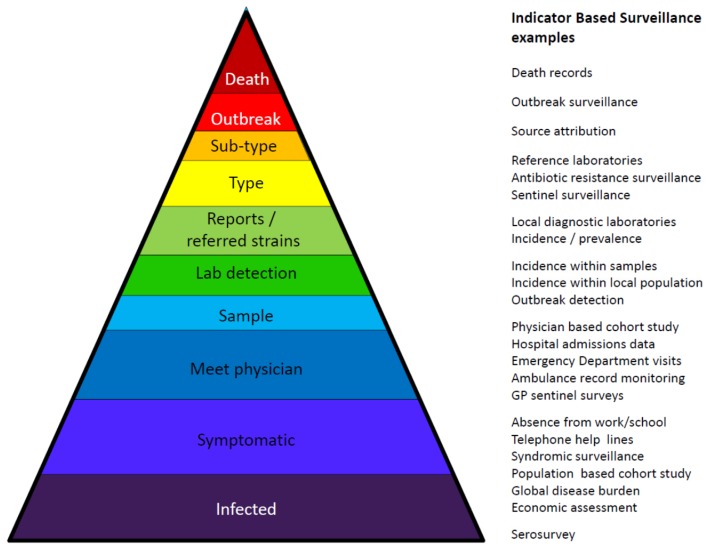
Surveillance pyramid and examples of the surveillance systems used to assess different parts of the pyramid *.

Notes: * The shape and distribution of the pyramid differs for each organism. Diseases and or pathogens in the ECDC TESSy database include AIDS, Antimicrobial resistance, Anthrax, Botulism, Brucellosis, Campylobacteriosis, Chlamydia infections, Cholera, Cryptosporidiosis, Diphtheria, Echinococcosis, Infection with Enterohaemorrhagic *E. coli*, Giardiasis, Gonococcal infections, Haemophilus influenzae, Hepatitis A, Hepatitis B, Hepatitis C, Infection with Haemophilus influenzae type b, HIV infection, Influenza, Legionellosis, Leptospirosis, Listeriosis, Malaria, Measles, Meningococcal disease, Mumps, Nosocomial infections, Pertussis, Plague, Pneumococcal infections, Poliomyelitis, Q-fever, Rabies, Rubella, Salmonellosis, Severe acute respiratory syndrome (SARS), Shigellosis, Smallpox, Syphilis, Tetanus, Toxoplasmosis, Trichinosis, Tuberculosis, Tularaemia, Typhoid/paratyphoid fever, Transmissible spongiform encephalopathies variant (CJD), Viral haemorrhagic fevers, Shiga/Verotoxin producing *E. coli* infection (STEC/VTEC), West Nile fever, Yellow fever, Yersinosis.

Surveillance is the on-going collection, validation, analysis and interpretation of health and disease data needed to inform key stakeholders and enable them to take action through planning and implementing effective, evidence-based public health policies and strategies for the control and prevention of diseases and epidemics [16,17]. Reported cases based on positive test results are often only the top of the surveillance pyramid (Figure 1). The degradation of information through the surveillance hierarchy remains a challenge, with detailed records that are somewhat unstructured at the individual physician level and highly structured surveillance records with limited data fields, less detail and, for some countries with a poor ability to examine the original records at national and, thus, at EU level. Surveillance data need to be timely and distributed to those who need it for the early detection and control of outbreaks, for measuring the impact of interventions, or for undertaking research. Surveillance may be compulsory or voluntary, active or passive, case-based or aggregated (although aggregated data is usually less useful). Some environmental surveillance data can also contribute to disease surveillance processes.

Ensuring that public health infrastructures are adequate is the best preparation for the coming changes in infectious diseases that will result from climate change and other drivers. It is therefore important to review existing surveillance systems and the data they provide as part of a response to these future risks. The purpose of this assessment is to review the current status of appropriate European monitoring systems. Here we examine the datasets they produce and assess their ability to monitor changes in infectious disease transmission and to pick up signals of new threats due to climatic and environmental change, as well as to identify potential weaknesses in their ability to detect climate change-related impacts.

This paper describes the infectious disease surveillance systems in place in the EU, cross surveillance initiatives and environmental surveillance data that can be used for investigating the environmental determinants of ID. The information sources are documented so that local, national and European public health practitioners and scientists can access these data to examine infectious disease epidemiology, evaluate intervention efficacy to look for impacts of climate and other change and to provide an evidence base for examining disease shifts and climate change adaptation initiatives.

## 2. Methods

The organisations, agencies, and networks involved in infectious disease surveillance in Europe were examined, along with their underlying legal framework, regulations, mandate and surveillance scope. Information on these organisations and networks, their current collaborations, the different surveillance systems and the environmental datasets was collected from surveillance experts, the peer-reviewed literature, grey literature and web sites of respective organisations, agencies and networks. Scientific and medical experts at the European Centre for Disease Prevention and Control were interviewed about the different surveillance systems maintained by the agency. Interviews were also held with a number of technical experts at other international agencies, including the European Food Safety Authority (EFSA). The methodology was predominantly descriptive, and designed to identify as many of the systems as possible. The ability of the different European surveillance systems to detect potential climate change signals was assessed, along with evaluations of how to best adapt these systems to identify new threats and changes in disease risks.

ECDC has developed the European Environment and Epidemiology (E3) Network with the goal of monitoring environmental precursors of epidemics and providing predictions that can be used for intervention [5]. The E3 Network has a group of experts in environmental epidemiology and a distributed, secure, web-based hub called the E3 geoportal [18]; that provides access to environmental datasets for assessing determinants of infectious and modelling outputs. European public health agencies and researchers can use this platform in preparedness and response to infectious disease spread in the short and long term such as environmental and social changes. The initial building-block of the E3 data repository was the data acquisition from the Emerging Diseases in a Changing European Environment project (EDEN), an FP-6 funded initiative. Further collaborations are on-going with several FP7 project in order to enrich the data repository. The repository is also used as a secure place to store project-specific geo-spatial data, such as the TigerMaps, DengueMaps or V-Bornet which generate novel geospatial data. The data files are re-classified into themes and categories and amendments to the metadata files are done to make the data more suitable for storage and maintenance in a database. Metadata standard for E3 data are formulated based on the mandatory elements of the INSPIRE metadata standards to the requirements of E3 on one hand, and ECDC-core metadata on the other hand. A set of metadata translation and compilation tools were developed to facilitate the authoring of metadata that complies with E3 standards. Contributors of data to the E3 Network can use these tools to author a compliant metadata file to accompany the data resources that they wish to submit to the E3 service. This tool is fully integrated into the E3 Geoportal. The environmental datasets cover a range of potential determinants of communicable diseases in the broadest sense: from past, current and future climatic parameters, landscape features, remote sensing information and socio-economic determinants that are known to have a key in human epidemiology (e.g., climate change datasets, land cover information, vegetation, hydrology, soil data, elevation, biota, wind speed; socio-economic data including population, economic, education, healthcare, hospitals, transport networks and statistics, migrant populations, demographic profiles, agriculture and livestock). Environmental datasets were identified and documented during the process of establishing the E3 Geoportal. Datasets from many sources were examined, and where relevant were stored for public access in the E3 Geoportal along with associated metadata. The data can be used in incident response, as a resource for investigation and to build understanding.

## 3. Results

### 3.1. Infectious Disease Surveillance Systems

Main infectious disease surveillance organisations, agencies and departments operating at the European level are documented (Table 1). Several of these are essential data sources for surveillance streams that can be used for examining the impacts of climate and environmental changes on geographical distribution, morbidity and mortality. Surveillance systems include both indicator and event based systems [17] and include data from many sources that can include mortality data, morbidity reports, laboratory data, outbreak data and field reports, vaccine and drug utilization, primary care surveillance (including sentinel systems), sickness absence data, syndromic surveillance *etc.* (Table 2).

European Union Member States (EUMS) have national surveillance systems using data from clinical (seldom used alone) and/or laboratory (e.g., salmonellosis) based systems, sentinel surveillance systems, in which only a proportion of practitioners or microbiologists report cases (e.g., influenza) or enhanced surveillance systems in which additional demographic and risk related data is collected (e.g., STEC/VTEC infection). The quality of data differs between EUMS, often by pathogen, due to differences in case definitions, the level of participation of data providers at different levels of the reporting systems (physician, hospital, laboratory diagnosis or laboratory reporting), technical equipment, and country-specific differences in health care systems organisation, surveillance infrastructure and public health capacity. ECDC has addressed these differences and is working to harmonize discrepancies through promoting disease networks and a common central health information system (TESSy).

**Table 1:** Main infectious disease surveillance organisations, agencies and departments operating at the European level.

Surveillance Organisation	Purpose
European Centre for Disease Prevention and Control (ECDC)	ECDC (established 2005) has the main goal of decreasing the incidence and prevalence of communicable disease in Europe. Article 3 of ECDC’s Founding Regulations describe the mission as to identify, assess and communicate current and emerging threats to human health from communicable diseases. ECDC works with national public health institutions across Europe to strengthen and develop Europe-wide disease surveillance and early warning systems, provides EU-level communicable disease surveillance, epidemic intelligence, early warning and response, and searches for, collects, collates, evaluates and disseminates relevant scientific and technical data, providing scientific risk assessments and opinions, scientific and technical assistance including training, timely information to the Commission, EUMS, Community agencies and international organisations active within the field of public health. It coordinates the European networking of bodies operating in the fields, including networks arising from public health activities supported by the Commission and operating dedicated networks for surveillance, information exchange, expertise and best practices, and facilitates joint actions. The current list of communicable diseases to be reported to ECDC by twenty-seven EUMS and three EEA/EFTA countries, Iceland, Lichtenstein and Norway, includes 52 diseases and conditions. [19]
World Health Organization (WHO) & WHO Regional Office for Europe	WHO, established in 1948, is responsible for international public health. WHO surveillance provides a portal to health statistics and detailed monitoring and assessment tools for key areas of health policy. WHO work in support of Member States’ surveillance, alert and response under the International Health Regulation (IHR) that came into force in 2007. IHR focal points report information on communicable diseases annually, particularly on vaccine preventable diseases, sexually transmitted diseases, hepatitis, communicable diseases and outbreaks, if the outbreak was reported under the International Health Regulations. WHO has surveillance programs of selected diseases [20].
World Organization for Animal Health (OIE)	Established in 1924, is an intergovernmental organisation responsible for improving animal health with 178 Member Countries and Territories who report information on animal diseases using immediate notifications and bi-annual and annual reports [21].
Food and Agriculture Organization (FAO)	Launched in 1945, is the main United Nations agency for food, and is mandated to secure enough high-quality food for all, improving agricultural, animal food production and the world economy. FAO engages in all aspects of agriculture production, fishery, food quality and food safety, and in all the different stages of food production.
Animal Production and Health Division (AGA)	AGA is FAO’s service for responding to animal disease emergences [22]. It includes the Animal Health Information Service (AGAH) Part of the Animal Production and Health Division (AGA) and is FAO’s source of technical expertise required for the rapid and effective control of trans-boundary disease emergencies. In the case of an animal disease emergency AGAH focuses on a combination of disease detection, early warning and response. These activities are carried out jointly with OIE [23].
Food Quality and Standards Service (AGNS)	Part of FAO committed to the enhancement of food safety and quality along the food chain at all levels, with the aim of preventing food-borne diseases and protecting consumers.
European Commission (EC)	The EC funds human surveillance systems through ECDC and research projects. Severe animal disease outbreaks are notified to the European Commission as well as to ADNS and OIE. The Commission coordinate several systems and platform to address public health threats and emergencies in the EU, including the network of the Early Warning and Response system [24]. DG-SANCO G2 coordinates notification of outbreaks in animals.
The European Food Safety Authority (EFSA)	EFSA was established in 2004 and is involved in the risk assessment of food and animal feed safety. EFSA works with national authorities and in consultation with stakeholders to provide scientific advice and communication on existing and emerging risks. EFSA examines data on zoonoses, antimicrobial resistance and food-borne outbreaks submitted by EUMS and produces EU Summary Reports [25].
Zoonoses Collaboration Centre (ZCC)	ZCC, EFSA and ECDC collaborate to produce the Annual Zoonoses Report [26].
European Environmental Agency (EEA)	Established in 1990 and operational in 1994, EEA is responsible for monitoring the European environment and publishes a five yearly assessment report “The European Environment State and Outlook”, with an overview of the environment in Europe. EEA works closely with EUROSTAT and collects and analyse different types of environmental data that are available to EUMS in a range of data bases and data sets, several of which are of interest in research on, and risk assessments of infectious diseases in the Region [27].

**Table 2:** Surveillance systems, tracking tools, communication systems, information exchange and outputs within Europe.

Surveillance Organisation /System/Tool	Type	Purpose
ECDC Epidemic Intelligence Information System (EPIS)	Event based threat tracking	EPIS is a secure communication platform tool, provided and coordinated by ECDC, that allows risk assessment bodies in the different European Members states (EUMS) to exchange non-structured and semi-structured information on current or emerging EU public health threats. It coordinates and shares surveillance and control work among national public health institutes to facilitate outbreak discovery. In public health crises, it allows epidemiological discussion among health institutes of the various Member States as well as political coordination based upon scientific conclusions). There are currently five different modules based on: Antibiotic Resistance and Hospital Acquired Infection (EPIS AMR-HAI), Sexually Transmitted Infections (EPIS STI), Food and Waterborne Diseases (EPIS FWD), *Legionella* Infections (EPIS ELDSNet) and Vaccine Preventable Diseases (EPIS VPD) [28].
ECDC Threat Tracking Tool (TTT)	Event based threat tracking	Event-based surveillance information is collected through epidemic intelligence activities on a daily basis, including a 24/7 duty system. Data are collected in an unstructured way and are studied, verified and primarily aimed at the detection of emerging threats. The data is collected in The Threat Tracking Tool (TTT) data base that allows ECDC to keep track of verified events with a known or possible impact on public health. ECDC uses the media, the web (ProMed, GPHIN, MediSys, *etc.*), specific websites (WHO, OIE, FAO, Governments, CDC, PH Institutes, *etc.*) and bulletins (Eurosurveillance, EpiNorth, MMWR *etc.*) for event-based surveillance [28].
European Surveillance System (see associated surveillance reports) (TESSy)	Routine EU surveillance	TESSy is the European database for collection, management and analysis of data on communicable diseases provided by the ECDC national contact points for surveillance. The system covers all statutory communicable diseases with the appropriate level of details, and follows EU-wide reporting standards, common principles of collaboration and agreements on data exchange, access and publication. Accurate and detailed systems are used to analyse surveillance data, provide trend analysis methods and models to identify subtle trends and low-level clusters or potential outbreaks [29].
Early Warning and Response System (EWRS)	Event based threat tracking	EWRS is a restricted network where Member States can alert other countries about serious outbreaks and severe diseases that could have an implication on other EUMS, and co-ordinate their responses. The ECDC Threat Tracking Tool is used to perform joint risk assessments where more than one Member State is affected [30].
ECDC Communicable Disease Threats Report (CDTR)	Event based threat tracking	The CDTR reports on communicable disease threats of concern to the European Union collated through epidemic intelligence activities are published weekly on the ECDC website [31].
Global Early Warning System (GLEWS)	Event based threat tracking	GLEWS combines and coordinates the alert and response mechanisms of OIE, FAO and WHO. The aim is to assist in the prediction, prevention and control of animal disease threats, including zoonoses, through information sharing, epidemiological analysis and joint field missions to assess and control the outbreak [32].
ProMED-mail (the Program for Monitoring Emerging Diseases) (ProMed)	Event based threat tracking	ProMed-mail publishes and transmits information on world-wide outbreaks of infectious diseases and acute exposures to toxins that affect human health, including those in animals and in plants grown for food or animal feed [33].
The Global Public Health Intelligence Network (GPHIN)	Event based threat tracking	GPHIN is a secure internet-based multilingual early-warning WHO-linked tool that continuously searches global media sources to identify informal information about disease outbreaks and other events of potential international public health concern [34].
Medical Information System (MediSys)	Event based threat tracking	MediSys is a tool initiated by the European Commissions (EC) Directorate General Health and Consumer Affairs (DG SANCO) for the purpose of supporting national and international surveillance networks in their monitoring of health-related issues of public concern, such as outbreaks of communicable diseases, bioterrorism, large-scale chemical incidents, *etc.* [35].
Emergency Prevention System for Transboundary Animal and Plant Pests and Diseases (EMPRES)	Event based threat tracking	EMPRES is a world-wide FAO programme. The strategy of EMPRES is to prevent and control diseases at source, across the food chain, including the occurrence of new emerging diseases [36].
International Food Safety Authorities Network (INFOSAN)	Event based threat tracking	A joint global network initiated by WHO and FAO that aims at rapid exchange of food safety issues, shared information, and provision of help to countries in need. INFOSAN collaborates with several of the other surveillance and response networks and systems [37].
The Global Foodborne Infections Network (GFN)	Event based threat tracking	GFN is a collaborative project of the WHO and a network of institutions and individuals world-wide with the purpose to detect, control and prevent food-borne and other enteric infections “from farm to fork”, with focus on inter-sectorial collaboration among human health, veterinary and food-related disciplines, and antimicrobial resistance in food-borne pathogens [38].
The Rapid Alert System for Food and Feed (RASFF)	Event based threat tracking	RASFF involves EUMS, the EU Commission and EFSA, and provides threat information about food and animal feed that could be a serious risk to human health (both microbiological and chemical threats) [39].
Eurosurveillance	Electronic Journal	Reports weekly on current health threats across Europe [40].
Communicable Disease Control in Northern Europe (EpiNorth)	Multi country Surveillance systems	EpiNorth project provides communicable disease surveillance, control and communication in the Nordic, Baltic countries and NW Russia. This includes case and outbreak surveillance (EpiWatch), news and events (EpiNews), an online journal (EpiNorth Journal), disease notification data (EpiNorthData), vaccination programmes (EpiVax), educational and training (EpiTrain) and links within networks (EpiLinks) [41].
Communicable Disease Control in The Mediterranean and the Balkans (EpiSouthNetwork)	Multi country Surveillance systems	EpiSouth Network aims at creating a framework of collaboration on epidemiological issues in order to improve communicable diseases surveillance and for enhancing communicable diseases surveillance and control of public health risks in South-East Europe, North Africa and the Middle-East [42].
Morbidity & Mortality Weekly Report (MMWR)	Electronic Journal	Reports weekly on current health threats in the US and other parts of the World [43].
WHO Centralized Information System for Infectious Diseases (CISID)	International surveillance	CISID is WHO/Europe’s main surveillance platform with information on communicable diseases, immunization coverage, and on recent outbreaks in Europe. It allows detailed reviews and assessments of infectious diseases in the WHO European Region and includes some subnational level data [44].
The European Health for All Database (WHO-HFA-DB)	International surveillance	WHO/Europe’s prime data source for international comparisons [45].
The Mortality database (WHO-MDB)	International surveillance	WHO data allows age- and sex-specific analysis of mortality trends by broad disease-groups, as well as disaggregated to specific causes of death dated back to 1980 [46].
The European Detailed Mortality Database (WHO-DMDB)	International surveillance	Provides mortality data by three-digit codes of the International Classification of Diseases, disaggregated by five-year age groups, back to 1990 [47].
European Hospital Morbidity Database (WHO-HMDB)	International surveillance	WHO data provides tools for the analysis and international comparison of morbidity and hospital activity patterns, based on hospital-discharge data by diagnosis, age and sex, back to 1999 [48].
EFSA-EU-wide baseline surveys	Food survey	The European Commission has organised baseline surveys on the occurrence of zoonotic agents in food and in various animal populations in the EU. EFSA is responsible for analysing and publishing the results of these surveys that will provide a knowledgebase for example for further considerations on specific control measures [49].
EUMS disease surveillance systems	National surveillance systems	Individual enhanced surveillance systems are organized for a number of key pathogens. For example a system of sentinel Dengue surveillance has been implemented in the Mediterranean region to monitor the emergence of autochthonous transmission.
Animal Disease Notification System (ADNS)	Animal surveillance	Animal diseases that EUMS are obliged to report are established through several pieces of legislation and are the responsibility of OIE (World organisation for animal health) and EU. Outbreak reports are sent by EUMS to the European Commission via the Animal Disease Notification System (ADNS) [50].
World Animal Health Information System (WAHIS)	Animal based threat tracking	WAHIS processes data on animal diseases in real-time and then informs the international community. WAHIS consists of an early-warning system and a monitoring system that monitors OIE listed animal diseases [23].
World Animal Health Information Database (WAHID interface)	Animal infectious disease surveillance	WAHID provides access to all data held within OIE’s World Animal Health Information System (WAHIS) [51].
Global Information and Early Warning System on food and agriculture (GIEWS)	Food based event threat tracking	GIEWS exchanges and analyses information about food production and security with other organizations, such as UN, governments, regional organizations, NGOs *etc.* and gets regular information from other early warning systems [52].
World Health Organization surveillance (WHO)	Worldwide disease surveillance	A portal to health statistics and detailed monitoring and assessment tools for key areas of health policy [53].
Triple S Project (SSS)	Syndromic surveillance	Started in Sep 2010 and co-financed by the European Commission SSS provides scientific and technical guidance for developing and implementing both human and animal syndromic surveillance systems, and produces an inventory of existing and proposed syndromic surveillance systems in Europe [54].
Global Alert and Response (WHO-GAR)	Outbreak alert and response	GAR is a global alert and response system for epidemics and other public health threats managed by WHO that helps EUMS to enhance epidemic preparedness, early warning alert and response [55].
Global Outbreak Alert and Response Network (GOARN)	Outbreak alert and response	WHO coordinates international outbreak technical response responses using resources from the Global Outbreak Alert and Response Network (GOARN) which was established in 2000 with the objectives of combating the international spread of outbreaks, ensuring that appropriate technical assistance reaches affected states rapidly and contributing to long-term epidemic preparedness and capacity building [56].
European Surveillance of Antimicrobial Consumption Network (ECDC ESAC-Net)	Drug consumption database	Pharmacies keep records of drugs sold both with and without prescription. ESAC-Net was established to provide representative national antimicrobial consumption data since 1997, which is useful for monitoring antimicrobial resistance across EUMS [57].
Network of medical entomologists and public health experts (Vbornet)	Vector borne disease surveillance	Produces distribution maps of the major arthropod disease vectors and related surveillance activities and defines priority strategic topics concerning the public health perspective of vector-borne diseases and vector surveillance [58].
European Union Summary Report on Trends and Sources of Zoonoses, Zoonotic Agents and Food-borne outbreaks in the European Union.	Report	Mandatory annual reporting currently involves eight zoonoses (brucellosis, campylobacteriosis, echinococcosis, listeriosis, salmonellosis, trichinellosis, tuberculosis (Mycobacterium bovis), Verotoxigenic Escherichia coli). Additional zoonoses and zoonotic agents may also be reported. Reports of suspected international outbreaks are collected, and analysed by EFSA and ECDC and presented annually in the EFSA Journal [59].
National telephone Help lines	Syndromic surveillance	Several countries use telephone help lines as an indirect indicator tool to detect outbreaks. This allows some large outbreaks of respiratory and enteric infections to be detected before sentinel and laboratory surveillance pick up the signal.
HealthMap	Event based threat tracking	Utilizes online informal sources for global disease outbreak monitoring and real-time surveillance of emerging public health threats. Media reports are incorporated and HealthMap is one of the main information sources for Epidemic Intelligence [60].
Global Atlas of Diseases	Interactive disease mapping	WHO’s Communicable Disease Global Atlas uses standardized WHO data and statistics for infectious diseases at country, regional, and global levels [61].
Drug sales	Surrogate surveillance	Drugs sold at pharmacies have been examined as sentinels for several food- and water-borne diseases which can give mild gastro-intestinal symptoms but may cause large undetected outbreaks, even if a large proportion of the population is affected.
Physician visits	Syndromic surveillance	Monitoring emergency department visits and patient visits to general practitioners are often used to detect outbreaks or increased risk of disease. Such information is available several days before results of microbiological sampling from patients. Monitoring increases in the occurrence of specific syndromes like gastrointestinal or lower respiratory symptoms are also possible.
Sickness records	Syndromic surveillance	Monitoring increased absence from work, schools and day care centres can be a tool for early detection of food- and water-borne outbreaks, influenza and lower respiratory infections.
Ambulance records	Syndromic surveillance	EUMS using rapid computerized reporting systems that monitor ambulance records can provide early information of increases in the occurrence of diseases/symptoms such as respiratory, gastrointestinal and influenza outbreaks.
Telephone surveys	Syndromic surveillance	In some EUMS Health Authorities contact people to elicit specific symptoms in order to detect the initiation of seasonal influenza increases.

Prior to the establishment of ECDC there were 17 dedicated (active) surveillance networks for various pathogens and some standardised case definitions. Historically, some of the surveillance data from different EUMS were not equivalent, representing as they were diverse diagnostic, laboratory and surveillance infrastructures as well as differences in prior exposure and infection rates within the EU community. Since European Centre for Disease Prevention and Control (ECDC) came into operation in 2005 region-wide surveillance data have been collected for over 52 notifiable diseases. For each notifiable disease a common standardized case definition has been agreed upon by the EUMS and ECDC, sometimes resulting in countries reporting data to ECDC that is different from that used at a national level. There is a central system for reporting notifiable disease and the case definitions and list of diseases is updated periodically.

Mandatory notification and laboratory surveillance are very effective in monitoring threats related to known risks. Such indicator-based surveillance will be able to show trends over time as well as changes in geographical distribution within the EU region, for example a spread of leishmaniasis or of tick-borne diseases and their vectors towards higher latitudes and altitudes due to a changing climate [62,63].

Event-based surveillance, on the other hand, focuses on recognizing new signals and emerging threats through the collection and study of unstructured data such as news releases, internet-based information and other epidemic intelligence sources. Outbreaks of non-notifiable diseases in an area will be observed through this type of surveillance as well as new threats. The emergence of wound infections in the northern countries around the Baltic Sea in the early/mid 2000s when several deaths occurred due to higher concentrations of non-toxigenic *Vibrio cholerae* in bathing waters after periods of unusually high water temperatures [64].

### 3.2. Laboratory Surveillance

Most of the common surveillance systems are based on laboratory surveillance, while mandatory reporting of some diseases by physicians occurs in some countries. Routine laboratory based surveillance may not be sufficient to detect emerging, re-emerging and new diseases and other types of surveillance are necessary, such as syndromic or sentinel surveillance, as well as surveillance of animal diseases, animal infections, environmental changes, drinking water and bathing water quality, food contamination *etc.* with increased collaboration between these reporting systems at a European level.

Both food and animal data are sometimes collected in a less systematic way than human disease data. Some EUMS have mandatory reporting (*i.e*., notification) for some or all reportable diseases both from laboratories and physicians and the number of physician reported cases are often not comparable with the number of confirmed laboratory reports for the same disease.

Pathogen specific surveillance is important for some pathogens that might be climate change related, and the pathogens that are most likely to be sensitive to climate change have been proposed [6]. Molecular surveillance uses the laboratory typing of pathogens to focus on a subset of pathogens and take action where there is an increase. ECDC initiatives on molecular surveillance are currently focusing on *Salmonella* and *Listeria* infections. An examination of long term trends in the impact of temperature on salmonellosis showed that this had changed over time and suggested that the impacts of climate change on different serotypes as a result of raised temperature have declined more recently [65]. There are also sequence databases focusing on organism phylogeny that can contribute to the understanding of human and animal diseases [66], but this paper has not reviewed these. There are also publications relating to climate change indicators [67], but these are not reviewed here.

There are a number of areas where classical surveillance may not capture all human infections [68]. Some pathogens are only commonly detected through cytology, histology, parasitology or haematology departments and reporting of infectious diseases from these may not be as complete as from diagnostic microbiology laboratories (e.g., *Pneumocystis jirovecii*; *Tropheryma whipplei*; *Enterocytozoon bieneusi*, *Plasmodium* spp. respectively) [69]. 

### 3.3. Syndromic Surveillance

Syndromic surveillance uses health-related information as a tool to monitor trends for any unexpected health outcomes and to detect outbreaks. This can sometimes be better for early detection of outbreaks such as seasonal influenza [70,71] and some environmental/climate related outbreaks. For example for the early detection of water-borne outbreaks after flooding events, by collecting data on over-the-counter sales of drugs, or calls made to telephone help lines [72,73]. An EU project called “TRIPLES” made an inventory of syndromic surveillance systems in place in Europe as well as proposing the development of a European platform for monitoring threats using syndromic surveillance data [74]. One of the well-known limitations of syndromic surveillance is that it is unspecific and can give false positive signals [75].

### 3.4. Sentinel Surveillance

The sensitivity of disease ID monitoring can be enhanced through sentinel surveillance where a rapid assessment of the incidence in certain area and during a certain season can be achieved. Designated sites are selected as sentinel institutions to represent a random sample of the population, in a certain area. Sentinel surveillance is useful for answering specific epidemiologic questions in a certain region, but may not represent the general population or the general incidence of disease, and may have limited usefulness in analysing national disease patterns and trends. Sentinel surveillance has been used for a long time to predict and follow increasing/decreasing trends during the influenza season [76]. A European system of sentinel dengue surveillance has been implemented in the Mediterranean region to monitor the emergence of autochthonous transmission [77]. Sentinel surveillance could be used to answer research questions such as the current distribution and incidence of a disease in a specific area, with follow-up studies examining changes over time and in space due environmental and/or climate change. For example, the incidence of Tick Borne Encephalitis (TBE) in an area could be studied by an on-going cross sectional sero-survey of all encephalitis patients that are admitted to a specific numbers of hospitals during a year, or by testing the blood of blood donors from a specific area, or by following annual seroconversion in a specified population. If this is only done for a short period this would be classed as a cross-sectional study. Positive serological results should be followed-up from an epidemiologic point of view, and could then be studied in relation to different determinants and drivers.

### 3.5. Cross-sectoral Surveillance

**Figure 2:**
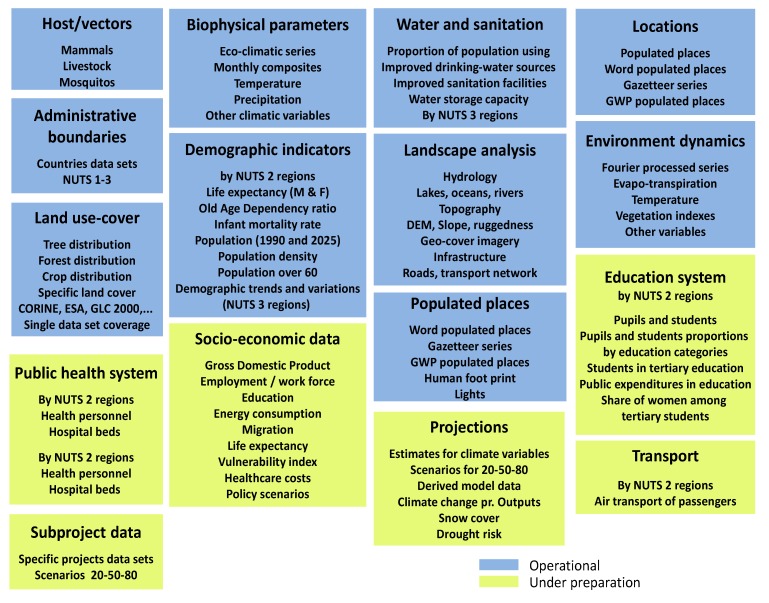
Datasets held in the E3 geoportal.

Surveillance collaboration between different sectors is useful for early detection of potential threats, or to assess changes in risk area distribution and in seasonal incidence. Collaboration between human case reporting systems at the national levels and within ECDC and other agencies/organisation (like EFSA, FAO, WHO, see Table 1) could be further strengthened. In addition, human infectious disease surveillance (Figure 2) benefits from collaborations with other sectors, such as the veterinary investigation of agricultural, domestic and wild animals, vector surveillance (e.g., VBORNET, see Table 3), water monitoring (drinking and bathing waters), food safety (“From the Farm to the Fork”), tourist industry and trade, travel information, health systems (including vaccination coverage), *etc.*

The Nomenclature of Territorial Units for Statistics (NUTS) is a hierarchical geocoding standard system for recording the geography and statistics of EU Member States. NUTS 0 are the member states; NUTS 1 are major socio-economic regions; NUTS 2 are basic regions for the application of regional policies and NUTS 3 are small regions [78].

**Table 3:** Datasets for Analytical Environmental Epidemiology.

Data Source	Short Description	Resource *(D:data, W:webservice)*	Spatial Cover
**Administrative Units**			
Eurostat (administrative boundaries)	The Statistical Office of the European Communities gathers and analyses harmonized data on demography and census information from the different European statistics offices. Eurostat maintains the Nomenclature of Units for Territorial Statistics (NUTS) used for delineating local, regional and national political and legal units that allows analytical links to population and census information maintain by Eurostat/GISCO geographical data. The NUTS subdivisions do not necessarily correspond to administrative divisions within the country [79].	D, W	Europe
Global administrative areas	The database provides the world's administrative boundaries at country and lower level subdivisions [80].	D	World
**Atmospheric Conditions**			
Advanced Very High Resolution Radiometer (AVHRR)	The AVHRR is a radiation-detection imager used to estimate mainly meteorological, hydrologic and oceanographic parameters including Sea Surface Temperature (SST), snow/ice cover and cloud covers. Data on the Earth’s surface observations ranges over more than 20 years [81].	D	World
AirBase	European air quality database provides data of networks and individual stations measuring ambient air pollution (notably particle matter PM_2.5_, PM_10_ and ozone). It is maintained by the European Environment Agency (EEA) through the European Topic Centre for Air Pollution and Climate Change Mitigation (ETC/ACM) and contains air quality data delivered annually under 97/101/EC Council Decision since 1997 [82].	D	Europe
Climate Research Unit (CRU)	The unit at the University of East Anglia provides global grids derived from interpolated meteorological station data (daily mean temperature, diurnal range, frost day frequency, precipitation, wet day frequency, and atmospheric pressure, circulation and drought indices) that have found extensive use in epidemiological studies [83].	D	Various/World
ENSEMBLES	High resolution, global and regional Earth System models developed in Europe, validated against quality controlled, high resolution gridded datasets for Europe, with probabilistic estimate of uncertainty in future climate at seasonal and decadal timescales [84].	D	Europe
European Reanalysis and Observations for Monitoring (EURO4M)	The European Reanalysis and Observations for Monitoring is an EU funded project (FP7) that provides information about the state and evolution of the European climate. The observation and reanalyses datasets of several atmospheric ECV’s have been developed as part of EURO4M are available through the project repository [85].	D	Project dependant
International Climate Assessment & Dataset (ICA&D)	The concept is to build a web data portal for daily station data and derived indices brought together under regional cooperation under the model used for the European Climate Assessment & Dataset (ECA&D) [86].	D	World
World Meteorological Organisation (WMO)	World climate data from the United Nations Statistics Division (UNSD) of the Department of Economic and Social Affairs (DESA) [87].	D	World
WorldClim	A set of global climate layers (climate grids) and coarse time frame (50 years, e.g., 1950–2000) for mapping and spatial modeling current conditions (interpolations of observed data, representative of 1950–2000), future conditions (global climate model (GCM) data from CMIP5 (IPPC Fifth Assessment)) and past conditions (downscaled global climate model output; projections for 2020, 2050 and 2080 at 1 km, 5 km, 10 km and 20 km resolutions) [88].	D	World
European Climate Assessment & Dataset (ECA&D)	ECA&D presents data and information on weather and climate extremes, together with daily datasets to monitor and analyse these extremes. It was initiated by the ECSN in 1998 and funded by EUMETNET and EC [89].	D	Europe
**Biodiversity and Bio-geographical Regions**			
Conservation Data	General data portal on Nature Conservancy’s core conservation datasets (land and water; terrestrial, marine and freshwater Eco-regions) [90].	D	World
Freshwater, marine and terrestrial Ecoregions (World Wildlife Fund)	An eco-region corresponds to “large unit of land or water containing a geographically distinct assemblage of species, natural communities, and environmental conditions” including the relative richness of freshwater, marine and terrestrial species [91].	D	World
**Elevation**			
ASTER Global Digital Elevation Model	ASTER GDEM is a World digital elevation model on a 1 arc-second (approximately 30 m at the equator) grid [92].	D	World
DEM of Shuttle Radar Topography Mission(SRTM V1 and V2)	NASA produces digital topographic data (SRTM version 2) that contains the vector coastline mask derived (SRTM Water Body Data (SWBD). Several open-source edited versions are available such as [93,94].	D	World
DEM of Shuttle Radar Topography Mission (SRTM V4)	The DEM of Shuttle Radar Topography Mission produced by NASA has been post-processed and improved (SRTM 90 m Digital Elevation Database v4.1 has resampled SRTM data to 250 m resolutions for the entire globe) [95].	D	World
DEM of the Global Land One-kilometer Base Elevation (GLOBE)	The DEM from the National Geographic Data Center at NOAA has a resolution of 30-arc-second (1 km) gridded [96].	D	World
Digital Elevation Model (DEM) of Europe	Digital elevation model (DEM) derived from GTOPO30 at EEA (elevation and hill shade) [97].	D	Europe
Digital Surface Model (DSM) of Europe	The EU-DEM a Digital Surface Model representing the surface as illuminated by the sensors, is a 3-D raster dataset with elevations captured at 1 arc second postings or about every 30 m [98].	D	Europe
**Environmental Monitoring Facilities**			
Aster data	The Advanced Space borne Thermal Emission and Reflection Radiometer (ASTER) is an imaging instrument on-board the Terra satellite and produces high spatial resolution data in 14 bands (visible to the thermal infrared) and digital elevation model [99].	D	World and specific product for USA.
Copernicus	Copernicus (previously Global Monitoring for Environment and Security) is an EC/ESA/EEA program aimed at developing European information services based on satellite Earth Observation and *in situ* data. Copernicus is covering six main thematic areas: Land Monitoring, Marine Monitoring, Atmosphere Monitoring, Emergency Management, Security and Climate Change [100].	W	Europe
Earth Observing System Data and Information System (EOSDIS)	Recent metadata and service discovery tool from NASA for discovering Earth Science data [101].	D, W	World
Environmental Marine Information System (EMIS)	EMIS contains information about biological and physical variables generated from both hydrodynamic models and satellite remote sensing in 2 dataset resolutions (4 km or 2 km) for several sensors at the Pan-European scale [102].	W	Europe
Euro Forest Portal (from EFI)	EFP contains information about forest information from the European Forest Institute. (EFI), whose mission is to carry out, strengthen, and mobilise forest research, expertise, policy and governance [103].	D	Europe
European Alien Species Information Network (EASIN)	The European Alien Species Information Network (EASIN) aims at improving the access to data and information on alien species in Europe [104].	D	Europe
European Forest Data Centre (EFDAC)	EU forest data and information (historical data, forest-related topic and European Forest Fire Information System (EFFIS) [105].	W	Europe
Fraction of Absorbed Photosynthetically Active Radiation (FARPAR)	Vegetation data monitoring program (The FAPAR quantifies the fraction of the solar radiation absorbed by live leaves for the photosynthesis activity) [106].	D	World
Institute for Environment and Sustainability (IES)	The Institute for Environment and Sustainability (IES) is one of the seven scientific institutes of the European Commission’s Joint Research Centre (JRC). The IES hosts many data portals and unique scientific databases (see specific topic in the table) [107].	D, W	Europe
Landsat 8	Landsat 8 satellite images are available at no charge (panchromatic band (black & white with resolution of 15 m, multispectral images with resolution of 30 m). True colour satellite image composites are often used as background information on maps replacing orthorectified aerial photographs [108].	D	World
MODerate Resolution Imaging Spectroradiometer (MODIS)	These MODIS data enhance the understanding of global dynamics and processes occurring on the land, in the oceans, and in the lower atmosphere. This is a key data source for epidemiological applications. MODIS (or Moderate Resolution Imaging Spectroradiometer) is a sensor aboard both Terra (EOS AM) and Aqua (EOS PM) satellites. The Earth’s surface is fully acquired every 1 to 2 days with 36 spectral bands at 250 to 1,000 m resolution [109].	D	World
Nansen Environmental and remote sensing center (NERSC)	The center focusses on monitoring and assessing regional climate change in high latitudes, with environmental datasets focusing on the arctic region (Ocean climate modelling, sea ice cover, forecast of ocean and sea ice, blooms and water quality, high resolution satellite radar) [110].	D	Arctic
NOAA Satellite and information service (NESDIS)	The National Oceanographic and Atmospheric Administration maintains the Satellite and Information Service with three data centres: the national oceanographic data center (NODC) for NOAA’s Ocean Data Archive, NOAA’s National Climatic Data Center (NCDC) provides climatological services and data and NOAA's National Geophysical Data Center (NGDC) providing long-term scientific data and geophysical data [111].	D, W	World
VITO distribution portal	Satellite images are recorded on the same platform and are coarse spatial resolution images with a very regular repeat cycle (almost daily), mainly used for vegetation related land cover [112].	D, W	World
**General Data Portal/Repository**			
Blue Marble Imagery	The Blue Marble corresponds to a mosaic of satellite images of the earth with the clouds removed (by month of 2004) [113].	D, W	World
Center for International Earth Science Information Network (CIESIN)	The NASA Socioeconomic data and application center and the Center for International Earth Science Information Network (CIESIN) at Columbia University is user with a series of social, natural and environmental data [114].	D	Various/World
Center for Sustainability and the Global Environment (SAGE)	The Center for Sustainability and the Global Environment (SAGE) produces synthesized satellite-derived land cover data (irrigated and urban areas) and agricultural census data to produce global data sets such as of the distribution of 18 major crops across the world [115].	D	Various/World
Community Data Portal (CDP)	The Community Data Portal (CDP) is a collection of earth science datasets from NCAR, UCAR, UOP, and participating organizations [116].	D	Various
Community Image Digital portal (CID)	Satellite remote sensing data archive and derived products hosted at the EU Joint Research Centre (JRC) [117].	D	Various
Data Distribution Centre (DDC) of the Intergovernmental Panel on Climate Change (IPCC)	The Data Distribution Centre (DDC) of the Intergovernmental Panel on Climate Change (IPCC) provides climate, socio-economic and environmental data, both from the past and under future scenarios (covering IPCC assessment report) [118].	D	World
E3 Network	ECDC has a European Environmental Epidemiology geospatial data repository (E3 Geoportal) for a wide array of spatial data archives for infectious disease epidemiology in Europe [18].	D, W	Europe/World
Emerging Diseases in a changing European eNvironmentEDENext—Biology and control of vectorborne infections in Europe (EDENext)	A large integrated EU funded research project (FP7) on Biology and control of vector-borne infections in Europe. The project focuses on investigating the biological, ecological and epidemiological components of vector-borne disease introduction, emergence and spread. This site the EDENext project host a Data Portal designed as a resource for the project partners posting spatial data, tools and links [119].	D	Europe
EuroGeoGraphics	EuroGeographics is an international non-profit association with 52 national mapping and cadastral agencies as members. EuroGeographics is providing users with four pan European geographic datasets: EuroDEM (ground surface topography), EuroBoundaryMap (EBM), EuroRegionalMap (ERM) and EuroGlobalMap (EGM). Eurographics is contributing to the European Location Framework aiming to build a geospatial reference data infrastructure and provides interoperable reference data and services from national information [120].	D	Europe
European Climate Adaptation Platform (CLIMATE-ADAPT)	The European Climate Adaptation Platform (Climate-ADAPT supported by EC and EEA) aims to support Europe in adapting to climate change. It is an initiative of the European Commission and helps users to access and share information on expected climate change in Europe, current and future vulnerability, adaptation strategies, case studies and tools [121].	D	Europe
European Environmental Agency (EEA)	The European Environment Agency (EEA) is the main independent information source on environment in Europe. Several datasets which are of interest for research and risk assessments of infectious diseases. Datasets include high resolution land cover maps relevant to risk analyses for vector and rodent-borne diseases. EEA also surveys water quantity and quality (indicators of microbiological pollution) in EUMS, with water sampling from around 20,000 bathing beach sites in fresh and coastal waters throughout the bathing season (May to September), under the EU Bathing Waters Directive [27].	D	Europe
European Observation Network for Territorial Development and Cohesion (ESPON)	The European Observation Network for Territorial Development and Cohesion (ESPON 2013 Programme) was adopted by the European Commission to support policy making related to regions and cities in Europe. The program is maintaining and expanding the ESPON 2013 Database focusing on territorial structures, with past, current situation and future trends of European territories with various geographical contexts and themes [122].	D	Europe
European Union Open data Portal	The site is run by the Communication department of the European Commission on behalf of the EU institutions [123].	D, W	Europe
European University Institute	EUI is an international research centre. The Economic and Social Data Portal provides access to a wide repository of macro and micro social variables notably from the European Social Survey (ESS) [124].	D	Europe
Geo Portal Group on Earth Observations-GEO	The Group on Earth Observations (GEO) is coordinating efforts to build a Global Earth Observation System of Systems (GEOSS) based on voluntary partnership of governments and international organizations. The GEO portal is gathering datasets derived from Earth Observations, including health impacts [125].	D	World
Geoland 2	This FP7 project is part of the Global Monitoring for Environment and Security (GMES) joint initiative of the European Commission and European Space Agency. It covers data such as land use, land cover change, soil sealing, water quality and availability, spatial planning, forest management, carbon storage and global food security. Core mapping services cover Land Cover and Land Use Monitoring Product, Biophysical Parameters and Seasonal Change [126].	W	Europe/World
GeoNetwork-FAO	The FAO GeoNetwork provides Internet access to interactive maps, satellite imagery and related spatial databases maintained by FAO and its partners. Information includes administrative boundaries, agriculture and livestock, base baps and topography, biological and ecological resources, soil resources, climate, fisheries, forestry, hydrology and water sources, land cover and land use/infrastructures, population, Socio-Economic Indicators and Human Health [127].	D, W	World
Global Change Master Directory (GCMD)	The Global Change Master Directory is one of the largest public data/metadata inventories which cover subject areas within the Earth and environmental science, The GCMD serves as an entry point for access to NASA Data sets, ancillary description, Services and tools with more than 29,000 Earth science data set and service descriptions [128].	D, W	Europe
Global Environment Outlook portal	The GEO Data Portal is the authoritative source for data sets used by UNEP and its partners in the Global Environment Outlook report and other integrated environment assessments [129].	D	World
GoGeo	EDINA delivers online Geospatial resources for education and research, services and tools to benefit students, teachers and researchers in UK [130].	D	World
GRID-Arendal	GRID-Arendal is a centre collaborating with the United Nations Environment Programme (UNEP) is hosting a gallery of maps and g graphics Library cataloguing graphic products from the last 15 years in a wide range of themes related to environment and sustainable development [131].	D	World
International steering committee for global mapping (ISCGM)	The International Steering Committee for Global Mapping (ISCGM) aims to examine measures that concerned national, regional and international organizations can take to foster the development of Global Mapping in order to facilitate the implementation of global agreements and conventions for environmental protection. The platform offer access to land cover and vegetation (cove percentage) [132].	D	World
IRI/LDEO Data Library	The International Research Institute (IRI) for Climate and Society maintain a large repository of climate, socio-economic, and geophysical datasets (data portal and map room) [133].	D, W	World
Organisation for Economic Cooperation and Development (OECD)	The Organisation for Economic Co-operation and Development (OECD) provide with a list of key socioeconomic indicators [134].	D	World
UNDATA	The United Nations Statistics Division (UNSD) of the Department of Economic and Social Affairs (DESA) is maintaining an internet based dataset service for the user community (themes: Agriculture, Crime, Education, Employment, Energy, Environment, Health, HIV/AIDS, Human Development, Industry, Information and Communication Technology, National Accounts, Population, Refugees, Tourism, Trade, as well as the Millennium Development Goals indicators) [135].	D	World
United Nations Development Programme (UNDP Europe Central Asia)	Socio-economic data from developing countries of eastern Europe and central Asia (Belarus, Kazakhstan, Kyrgyzstan, Moldova, Russia, Tajikistan, Turkey, and Ukraine) [136].	D	Europe and Central Asia
Untied Nations Department of Economic and Social Affairs (UN DESA)	United Nations Department of Economic and Social Affairs (Population division) maintains datasets on population trends, urban/rural population, international migrant stock, global migration database and other socio-economic parameters [137].	D	World
Vector Map Level 0 (VMap0) and Vector Map Level 1 (VMap1)	The Vector Map Level 0 (VMap0—low resolution) and Level 1 (VMap1—medium resolution) databases are designed to provide vector-based geospatial data representing six continental regions of the world. Vmap0 can be ordered (four CDs) [138].	D	World
WorldMap	The WorldMap open source platform is being developed by the Center for Geographic Analysis (CGA) at Harvard University to explore, visualize, edit, download and publish geospatial information. A wide collection of resources are available under the WorldMap data repository [139].	D	World
**Geographical Names**			
Geonames	Geographical database on place names in various languages [140].	D	Europe
**Geology and Soil**			
One Geology Europe	OneGeology-Europe aims to create a dynamic digital geological map data for Europe Geological datasets [141].	D	Europe
European Soil Portal	Under The Land Resource Management Unit at Institute for Environment and Sustainability (JRC), ESP contains digital resources grouped in data, maps and application/services on soil information; at European scale, while, when possible, links to national or global datasets [142].	D, W	Europe
**Habitats and Biotopes**			
Anthropogenic biomes of the world	Anthropogenic biomes delineate human influence on global ecosystems integrating human and ecological systems released in 2008 by Ellis and Ramankutty [143].	D	World
**Human Health and Safety**			
Atlas on water and health (V2)	The atlas provides information about indicators related to health, water, and sanitation using country-wide data on a yearly basis from various source (Joint Monitoring Programme, Centralized Information System for Infectious Diseases, World bank, UNDP ....). It is maintained by Institute for Hygiene and Public Health, WHO Collaborating Centre for Health Promoting Water Management and Risk Communication (IHPH) [144].	D	WHO European countries
**Hydrography**			
Catchment Characterisation and Modelling (CCM)	River Basins, Catchments and Rivers for Europe maintained by Institute for Environment and Sustainability (IES) at the Joint Research Center (JRC) [145].	D	Europe
Global Lakes and Wetlands Database (GLWD)	The nature conservation organization World Wide Fund for Nature (previously named World Wildlife Fund) provides access to a World Global Lakes and Wetlands Database (GLWD). The Level 1 (GLWD-1) corresponds to lakes (area ≥ 50 sq. km) and largest reservoirs, the Level 2 (GLWD-2) permanent open water bodies with a surface area ≥ 0.1 sq. km excluding the water bodies contained in GLWD-1 and Level 3 (GLWD-3) all lakes, reservoirs, rivers and different wetland types in the form of a global raster map at 30-second resolution. Access Level 3 data: For GLWD-3, the polygons of GLWD-1 and GLWD-2 were combined with additional information on the maximum extents and types of wetlands. Class “lake” in both GLWD-2 and GLWD-3 also includes man-made reservoirs, as only the largest reservoirs have been distinguished from natural lakes. It draws upon existing maps, data and information, producing new data which contains the best available sources for large lakes and reservoirs, smaller water bodies and wetlands, and was developed in partnership with the Center for Environmental Systems Research, University of Kassel, Germany [146].	D	World
Global Water Scarcity Information Service (GLOWASIS)	GLOWASIS is a collaborative European FP7 project aimed at pre-validation of a GMES Global Water Scarcity Information Service in combining hydrological models and in-situ and satellite derived water cycle information [147].	D	Europe
International Water Management Institute (IWMI)	IWMI produces the global irrigated area map and associated products (global map of irrigated area, Global map of Rained Cropped Areas, Global map of all land use/land cover and areas) based on using multiple satellite sensor and secondary data [148].	D	World
Water Information Systems for Europe (WISE)	The Water and Information System for Europe is a partnership between the European Environment Agency and the European Commission. The aim is to deliver data from the Bathing Water Directive from around 20,000 bathing sites in the EU Region and results are presented in a “Quality of bathing water” annual report published by EEA and the EC [149].	D	Europe
**Land Cover**			
Global Land Cover Characteristics (v2.0)	The global land cover characteristics database was developed on a continent-by-continent basis with 1 km nominal spatial resolution, and is based on AVHRR data (April 1992–March 1993). The version 2.0 of the Global Land Cover Database contains updated land cover and water classes [150].	D	World
Corine Land Cover data (2000)	The Corine inventory databases and several of its programmes have been taken over by the European Environment Agency (EEA). This database land cover is operationally available for most areas of Europe (44 classes and scale of 1:100,000). Produced by EEA the with IMAGE2000 products have been used for updating the European land cover database and are primarily derived from Landsat 7 Enhanced Thematic Mapper (ETM) 7 imagery, and are georeferenced and orthorectified, making them high quality and of relevance for landscape epidemiology of vector- and rodent-borne diseases [151].	D	Europe
Corine Land Cover data (2006)	Corine land cover 2006 is the first CLC database (vector files) update which was finalised in the early 1990s as part of the European Commission programme to Coordinate Information on the Environment (Corine).This database was processed by The European Topic Centre on Land Use and Spatial Information and owned by EEA [152].	D	Europe
ESA GLOBCOVER V2	The GlobCover Land Cover using satellite imagery product is European Space Agency initiative in collaboration with EEA, FAO, GOFC-GOLD, IGBP, JRC and UNEP. The Global Land Cover Map provides users with information relevant to land use, ecosystems and climate change (80 land cover categories included) [153].	D	World
Global Land cover 2000 (GLC 2000)	Land cover (what covers the surface of the Earth, e.g., grass, forest, urban) and land use data (often derived from land cover data to indicate the function of the land, e.g., agriculture) are terms that are often interchanged because they can have overlapping classes, e.g., forests represent land cover and land use. The Global Land cover 2000 (GLC 2000) is a harmonized World land cover database over the whole globe of year 2000. The GLC 2000 is based on 14 months of pre-processed daily global data acquired by the VEGETATION instrument on board the SPOT 4 satellite [154].	D	World
Image 2000 database	Image2000 products are the main source of data for updating the European Land Cover database (CORINE Land Cover), but are also reference data in themselves. Primarily derived from Landsat 7 Enhanced Thematic Mapper ETM+imagery, are georeferenced and orthorectified, resulting in a consistent, high quality product [155].	D	Europe
TrueMarble^TM^	The True Marble global dataset is lower resolution satellite imagery for the earth with clouds removed under a Creative Commons Attribution [156].	D	World
**Natural Risk Zones**			
Dartmouth Flood Observatory	The Dartmouth Flood Observatory is archiving digital map record of surface water and conduct remote sensing-based measurement [157].	D	World
European Drought Observatory (EDO)	The EDO at the Joint Research Center (JRC) contain of drought indicators derived from different data sources (e.g., precipitation measurements, satellite measurements and modelled soil moisture content) [158].	W	Europe
European Floods Portal (EFP)	The European Floods Portal (EFP) at the Joint Research Center (JRC) brings together information on on-going and forecasted river floods and flood risk in Europe [159].	W	Europe
**Oceanographic Geographical Features**			
Global Marine Information System (GMIS)	The GMIS WMS about on Earth Observation data, derived from optical and infrared satellite sensors are accessible in 2 dataset resolutions (4 km or 9 km) for several sensors (MODIS-AQUA, SEAWiFS, MERIS, PATHFINDER) at the global, Africa, Pacific and Caribbean geographical coverage [160].	W	Europe
Global Multi-resolution Topography Data Portal (GMRT)	The Global Multi-Resolution Topography (GMRT) synthesis is a multi-resolution compilation of sonar data collected World [161].	D, W	World (ocean)
Oceancolorweb	Satellite data products from NASA's ocean observation [162].	D	World (ocean)
**Population Distribution—Demography**			
Country-level Downscaled Population and Income Data	The Center for International Earth Science Information Network propose a country-level population and downscaled GDP projections based on the B2 Scenario, 1990–2100 [163].	D	Various/World
Geostat	The GEOSTAT 2006 dataset is a European population grid dataset for the reference year 2006 at 1 sq. km resolution. It contains the total population of the four EFTA countries and all EU countries, with the exception of Cyprus for which no LAU2 population data were available for the reference year 2006. Users may freely copy, publish and distribute the GEOSTAT 2006 grid dataset within their own organisation (company, governmental authority, municipality) [164].	D	Europe
GISCO	A Eurostat GIS service of the European Commission, which provides administrative and statistical units, area management/restriction/regulation zones and reporting units, land cover and Urban Morphological zones, population distribution, the degree of urbanization and transport networks (airports, ferry lines, ports, roads and railways) [165].	D	Europe
Global Rural-Urban Mapping Project, Version One (GRUMPv1)	World high resolution gridded population data based on satellite measurement and census data published in 2011 [166].	D	World
Gridded Population of the World (GPWv3 and GPW fe)	The gridded population of the world is a World population data estimates provided for 1990, 1995, and 2000 (GPW V3), and projected to 2005, 2010, and 2015 (GPW fe). The latter product was produced in collaboration with the United Nations Food and Agriculture Programme (FAO). The product provides user with both available for population count and density per grid cell at various resolutions (2.5 arc-min (5 km at the equator), 1/4 degree, 1/2 degree and 1 degree) useful for analysis with social, economic, and earth science data [167].	D	World
Human Footprint Dataset	The Wildlife Conservation Society (WCS) and the Center for International Earth Science Information Network (CIESIN) at Columbia University has joined together to systematically map and measure the human influence on the Earth’s land surface: map of wild areas, Global Human Influence Index (IGHP) and Global Human Footprint [168].	D, W	World
**Protected Sites**			
World Database on Protected Areas (WDPA)	The World Database on Protected Areas (WDPA) corresponds to a global dataset on marine and terrestrial protected areas from multiple sources. It is a joint venture produced by UNEP-WCMC and the IUCN World Commission on Protected Areas (IUCN-WCPA) working with governments and collaborating NGOs [169].	D	World
World Intact Forest Landscapes	The World database of Intact Forest Landscape is based on a global assessment of intact forest landscapes based on available satellite imagery. This corresponds to areas of forest landscapes larger than 500 km^2^ that are fragmented by roads, settlements, waterways, pipelines and power lines [170].	D	World
**Species Distribution**			
Gridded Livestock of the World (GLW)	The resource correspond contemporary global distribution maps for the main species of livestock (cattle, buffaloes, goats, sheep, pigs and poultry) [171].	D	World
Livestock Geography Atlas Global Livestock Production and Health Atlas (GLiPHA)	The interactive atlas is aiming to present global animal production and health statistics (map, table and graphics) from the Animal Health and Production Division (AGA) at FAO [172].	D	World
**Transport networks**			
Global Roads Data	The Global Roads Open Access Data Set (gROADS) is public domain global road map supported by the International Council for Science’s Committee on Data for Science and Technology (ICSU-CODATA). [173].	D	World
OpenStreetMap	OpenStreetMap emphasizes local knowledge in order contribute and maintain data and global map (roads, trails, railway stations, land use) World [174].	D	World
World Port Index	The world-wide database contains the location and characteristics of major ports and terminals (tabular format and GIS files) [175].	D	World
**Utility and Governmental Services**			
Global Disaster Alert and Coordination System (GDACS)	GDACS is a cooperation framework between the United Nations (UNOSAT and OCHA), disaster managers and the European Commission aiming at filling the information and coordination gap in the first phase after major disasters with Event-based data, map and satellite imagery [176].	D, W	World
UNITAR’S Operational Satellite Applications Program (UNOSAT)	UNITAR’S Operational Satellite Applications Program (UNOSAT) is a programme delivering imager analysis and satellite solutions to UN and development organisations under the scope of humanitarian relief, human security, strategic territorial and development planning [177].	D	World

Collaboration on surveillance between human and veterinary sectors occurs at local, national and international level (e.g., investigation of STEC/VTEC outbreak in Germany; reporting of Highly Pathogenic Avian Influenza poultry outbreaks to the Commission) to detect changes in zoonotic disease risk, in combination with vector surveillance in areas where land cover/land use and climatic conditions are, or will become favorable for disease transmission. To ensure cross-sectoral surveillance activities, information to local stakeholders and actors are important to initiate and increase active participation.

Data on food, water and the environment that derive from disease control programs and process monitoring are important in preventing human health threats. This includes official controls, surveillance and other monitoring that are used to covering all stages of production, processing and distribution of food, together with information to the public of any risks to health. Regulation 178/2002/EC [178] states that food and animal feed on sale in EUMS should be safe. Food businesses are responsible for ensuring that their food and animal feed fulfils legal requirements and are checked by food authorities related to the producer or at import from non-EU states. There is free movement of foods within Europe and food checked by local food authorities does not normally need to be re-checked. Unsafe food is withdrawn from the market and public warnings issued, with information reported to the Rapid Alert System for Food and Feed (RASFF) which provides food control authorities with a means for exchanging information about serious risks from food or feed.

Incidents where there is microbiological contamination of foods with *Salmonella*, *Campylobacter*, *Listeria monocytogenes*, verotoxigenic *E. coli*, and *Yersinia*, are also reported. The specific nature of molecular typing systems (e.g., Salmonella) can mean that the isolation of a pathogen from a food product can be used to link to identical isolates detected in a number of patients. The increasing use of typing based on whole genome sequencing may make raw food monitoring more productive in attributing pathogens detected to source food animals and transmission pathways. EUMS use different types of surveillance and monitoring to detect food-borne outbreaks. It is crucial that a food-borne outbreak is detected immediately in order to protect members of the public from preventable diseases. EUMS with suspected international outbreaks can communicate through the EPIS secured network at ECDC to report food-borne outbreaks on both mandatory and optional bases to EFSA. The aim is to follow trends, detect deviation from trend and examine emerging public health risks from new agents and food items. EFSA and ECDC are collecting this information and present it annually in the European Union Summary report on trends and sources of zoonoses, zoonotic agents and food-borne outbreaks in the European Union [179].

### 3.6. Environmental Surveillance

Environmental surveillance has been widely used for the detection of disease outbreaks, in disease reduction and as indicators for early warning systems and for source attribution [180,181,182,183,184,185,186]. The institution and data source platform are summarized into the Table 3.

The European Drinking Water Directive [187] on the quality of water intended for human consumption state that EU Member States must monitor potable water and take action if contaminated. EUMS can decide themselves if they want to include monitoring of private water sources as well. The monitoring of potable waters for *Cryptosporidium* oocysts in the UK has, for example, resulted in the early detection of outbreaks of cryptosporidiosis, in some cases with oocyst detection in water before the start of the outbreak [188]. Monitoring and control of oocysts in potable waters has resulted in significant reductions in cases of human cryptosporidiosis [189].

The E3 network aims to facilitate collaborative initiatives through the compilation and processing of environmental datasets, correlation and advanced analysis, supporting risk assessments and the rapid detection of emerging public health threats related to environmental factors. Previous work has included malaria, tick borne encephalitis (TBE) and vibriosis. Europe has since 2006 also had a Bathing Water Directive [190] that obliges EUMS to monitor bathing waters. The directive covers all types of surface waters (coastal and inland areas) where a large number of people are bathing.

## 4. Discussion

### 4.1. Use of Surveillance Data to Detect Changes in Threats, Studying Causes and Drivers, Project Changes in Risks, and Develop Adaptation Tools

Surveillance is, as described above, primarily used to detect changes in threats; either an increase in outbreak frequency, changes in seasonal incidence, changes in geographical risk distribution, or the introduction of new pathogens and/or disease vectors into new areas.

Surveillance data can also be used to study relationships with different determinants and drivers, such as climatic, environmental, socio-economic or demographic factors to better understand causes of observed changes. This is often done either by analyzing times series of reported cases, (detecting outbreaks retrospectively or prospectively) and comparing them to times series of exposure parameters or in seasonal incidence over time in an area, by in-depth studies of a specific outbreak, or by studying current differences between geographical areas if reliable historical data is not available. Outbreak surveillance is for example useful in the area of waterborne diseases [191], and the relations between rainfall and outbreaks has already been examined [192,193,194]. Both heavy rainfall and periods of sustained low rainfall appear to be associated with outbreaks. Similarly, cholera outbreaks have been analysed to examine global differences in seasonality [195].

Satellite and other remote data sources are useful tools when studying links between environmental and disease datasets over a larger geographic area, like the whole of Europe for example (Table 3). Satellite and remote sensing have been used to monitor and develop early warning systems for disease outbreaks and are being tested for some diseases world-wide [196]. Cholera outbreaks around the Gulf of Bengal can for example be projected by combining satellite monitoring of sea water surface temperatures and chlorophyll concentrations near the coast (indicating algal blooms in nutrient waters). Early-warning systems at a local level are, on the other hand, usually based on observed local data instead of satellite data. The Czech Republic has developed an early warning system for the risk of tick-borne diseases (TBE and Lyme borreliosis) based on a combination of known vector distribution (based on data from continuous vector surveillance), the ecology of tick activity, and weather forecasts over the coming week [197].

Disease vector models, for example, are often based on a combination of satellite data or local land cover/land use and climatic data, vector surveillance data, known vector ecology, and outcomes of climate change scenarios for the region. A country-based model on the northern spread of Lyme borreliosis in Sweden over the coming decades has been constructed based on a combination of vector surveillance data, tick ecology, local land cover and local climate change scenarios [198]. Accordingly, satellite data was the basis for models on possible distribution changes in Europe due to climate change of the Asian tiger mosquito, *Aedes albopictus*, the main vector of dengue fever and chikungunya fever in Europe [199]. Projections about future changes in disease risks due to environmental or climate changes can be made based on surveillance data in combination with temporal and geographic models derived from known epidemiological and ecological factors related to certain diseases. Such projections can either be made on a local scale or for a whole region. Projections that are applicable across countries are often based on satellite images and remote sensing in addition to other data.

Risk assessments of possible future changes in infectious disease risk from climate change in combination with other disease drivers [3] can be made either based on mathematical scenario models like the ones described above, or through theoretical models based on surveillance data and projections [200]. Adaptation measures and tools can then be developed in collaboration with local stakeholders and policymakers [200].

### 4.2. Access to Data

The examination of human disease against environmental data has a number of limitations that can make it difficult to conduct useful analysis. Not all the sources described in the tables provide easy on-line access to data, and some are covered by legal stipulations or commercial limitations. Human infectious disease data is subject to confidentiality and data security rules. Environmental data can be subject to problems including format, temporal and geographic resolution, completeness, and period covered, while human disease data can also be limited by temporal and geographic resolution as well as lack of demographic identifiers, risk markers and molecular typing data. There are also mapping issues in linking vector and raster based data. The development of geoportals to facilitate easy access to environmental data should improve access, and the ECDC E3 geoportal [18] developed for use with infectious diseases should improve this. For TESSy data there have been standards for reporting to provide comparable datasets and access rules to share data, but there is still diversity in the temporal basis of the report (e.g., onset, specimen, lab report, reporting date).

### 4.3. Completeness and Consistency of Human Disease Data

The human disease datasets are subject to variations in quality, and results can differ substantially between countries, both with regard to how the data is collected, what temporal and geographic markers are reported and the ascertainment level, that reflects differences in the whole chain from patient to physician to laboratory to surveillance reporting. Diagnostic and typing methodology can differ between laboratories and countries. Large studies using data from across Europe can allow interesting approaches to analysis, but TESSy data may be available for only a few years, and for most countries only at country level (NUTS 0). While national datasets can be accessed that extend over longer timescales these can require effort and agreement to establish for many countries.

### 4.4. Completeness and Consistency of Environmental Data

Many of the environmental datasets are obtained from remote satellite observation of the earth and their outputs are based on algorithms that are subject checked by ground observation data. Data can be missing because of cloud cover and many of the datasets are corrected for this using interpolation from adjacent geographic and temporal readings. Some datasets are more readily accessed than others, and there can be considerable differences in the temporal and geographic resolution of different types of data. The satellites providing datasets change over time and this may affect data quality, but the quality across Europe is thought to be good.

## 5. Conclusions

There are a range of organisations, institutions, systems and other tools involved in infectious disease surveillance in Europe at both national and EU regional levels. The quality and consistency of the data that these produce could in many cases be further improved. Increased collaborations between systems and across sectors as well as standardized definitions and methodologies would allow data to be analysed between locations and over time. Early signals of changes in disease burden and in geographical distribution as well as the introduction of new threats into the EU region due to environmental and climatic changes would be easier to pick-up and analyse in order that EUMS can develop adequate response measures.

Linking geographic information with infectious disease surveillance data can in many cases lead to a better understanding of the disease epidemiology in general and the impact of climate change in particular. This will require a more detailed understanding of the infectious disease drivers and how they interact [3]. Infectious disease data from national, expert and EU reference and surveillance systems such as TESSY data should provide the evidence base. Human case data need to be collected in a consistent way, while keeping the confidentiality of patient data. It should include parameters e.g., date of onset/specimen/ reporting/outbreak, and geographic location of infection. This should allow better linkage to different satellite derived variables, e.g. climate variables, land cover/land use data, vegetation index, and demographic data, as well as observed data on other relevant variables and drivers (Figure 2) depending on the eco-epidemiology of the specific infectious disease that is under study. The EU Member States will benefit from the results of such regional analyses by increased information about changes in geographical distributions, seasonality, disease burden, risk populations and possible new threats in different parts of the EU region. This can inform policy makers and intervention strategies.
